# Digital Health Integration Assessment and Maturity of the United States Biopharmaceutical Industry: Forces Driving the Next Generation of Connected Autoinjectable Devices

**DOI:** 10.2196/25406

**Published:** 2021-03-18

**Authors:** Ramin Rafiei, Chelsea Williams, Jeannette Jiang, Timothy Dy Aungst, Matthias Durrer, Dao Tran, Ralph Howald

**Affiliations:** 1 SHL Medical Zug Switzerland; 2 Department of Pharmacy Practice MCPHS University Worcester, MA United States

**Keywords:** digital health, artificial intelligence, drug delivery, biopharma, autoinjector, injectable devices, disease management, autoimmune, oncology, rare diseases

## Abstract

Autoinjectable devices continue to provide real-life benefits for patients with chronic conditions since their widespread adoption 30 years ago with the rise of macromolecules. Nonetheless, issues surrounding adherence, patient administration techniques, disease self-management, and data outcomes at scale persist despite product design innovation. The interface of drug device combination products and digital health technologies formulates a value proposition for next-generation autoinjectable devices to power the delivery of precision care at home and achieve the full potential of biologics. Success will largely be dependent on biopharma’s digital health maturity to implement this framework. This viewpoint measures the digital health maturity of the top 15 biopharmaceutical companies in the US biologics autoinjector market and establishes the framework for next-generation autoinjectable devices powering home-based precision care and the need for formal digital health training.

## Introduction

The interface of drug device combination products (DDCPs) and digital health technologies is rapidly expanding to provide new and innovative ways to improve a patient’s health care outcomes. DDCPs are therapeutic and diagnostic products that combine drugs, devices, or biological products and include prefilled syringes or autoinjectors. The digital health frontier is manifesting in many forms, such as software as a medical device, regulated wearable devices, and telemedicine and remote patient monitoring [1]. Although many definitions of digital health have been published, overall, these definitions encapsulate empowering patients and providers with technology that can lead to scalable medical care leveraging novel digital tools [2-4]. Progress toward embracing digital health has been sporadic over the past decade, and biopharmaceutical companies are no exception [5]. As they adopt digital health, they will also need to account for the inevitable shift of the US health care environment as it gravitates toward treatment in the home, focus on patient preferences, expanded outcomes, biosimilar adoption, and broader value-based care agreements [6,7]. Sophisticated digital health technologies can measure and monitor patient outcomes, address gaps in patient care, and support medication optimization; however, demonstration of their value will require the generation of clinical, economic, and usability evidence using data resources, predictive analytics, expanded endpoints (eg, digital biomarkers), and behavioral sciences, often superseding conventional models [8].

Digital health is already demonstrating potential when combined with DDCPs [9]. Autoinjectable devices have provided real-life benefits for patients in terms of drug self-administration, since their widespread adoption began 30 years ago with the rise of macromolecules for chronic conditions in the autoimmune space [10]. The share of biological products, many of which could be administered by autoinjectors, is growing in the US market, and they accounted for more than one-fourth of all new molecular entities approved (2015-2019) [11]. The evolution of how these drugs are delivered to patients has enabled companies to capture and drive market share and to create high brand loyalty. In recent years, there have been considerable shifts of design to enable patients to more easily utilize the device in their home setting by reducing the number of steps for activation and self-administration [12,13]. Nonetheless, issues surrounding adherence, patient administration techniques, disease self-management, and data outcomes at scale still persist despite product design innovation, and these are the next areas to be explored [14-18]. Arguably, this is a systems-based issue that affects multiple stakeholders beyond biopharma and is yet to be adequately addressed and solved.

Patients with chronic conditions are estimated to be nonadherent to their medications 25%-50% of the time, and those requiring home injections are no exception [19-25]. Majority of these biologics are administered via autoinjectors, which help improve patient adherence and are preferred for subcutaneous self-administration [26]. Research has identified that patients who are nonadherent to their autoinjectors incur high health care spending and exhibit further disease progression [22,26]. Digital health has been explored as a possible solution to this problem [27]. Biopharma has been an advocate for integrating digital technologies to address nonadherence, but there has been a considerable lack of advancement when looking at the injectable space. This slow pace of digital health adoption has often been attributed to regulatory barriers [28], although federal regulators are redefining their models for the evaluation of digital technologies, facilitating adoption [29].

Considering the expanse of the biologics market, the popularity of autoinjectors for patient self-administration, and the potential of digital health technologies advancing the role of autoinjectors in chronic disease management, we evaluated biopharma’s digital health maturity as an enabler of next-generation autoinjectable devices.

## Analysis of Digital Health Maturity in Biopharma

**Figure 1:**
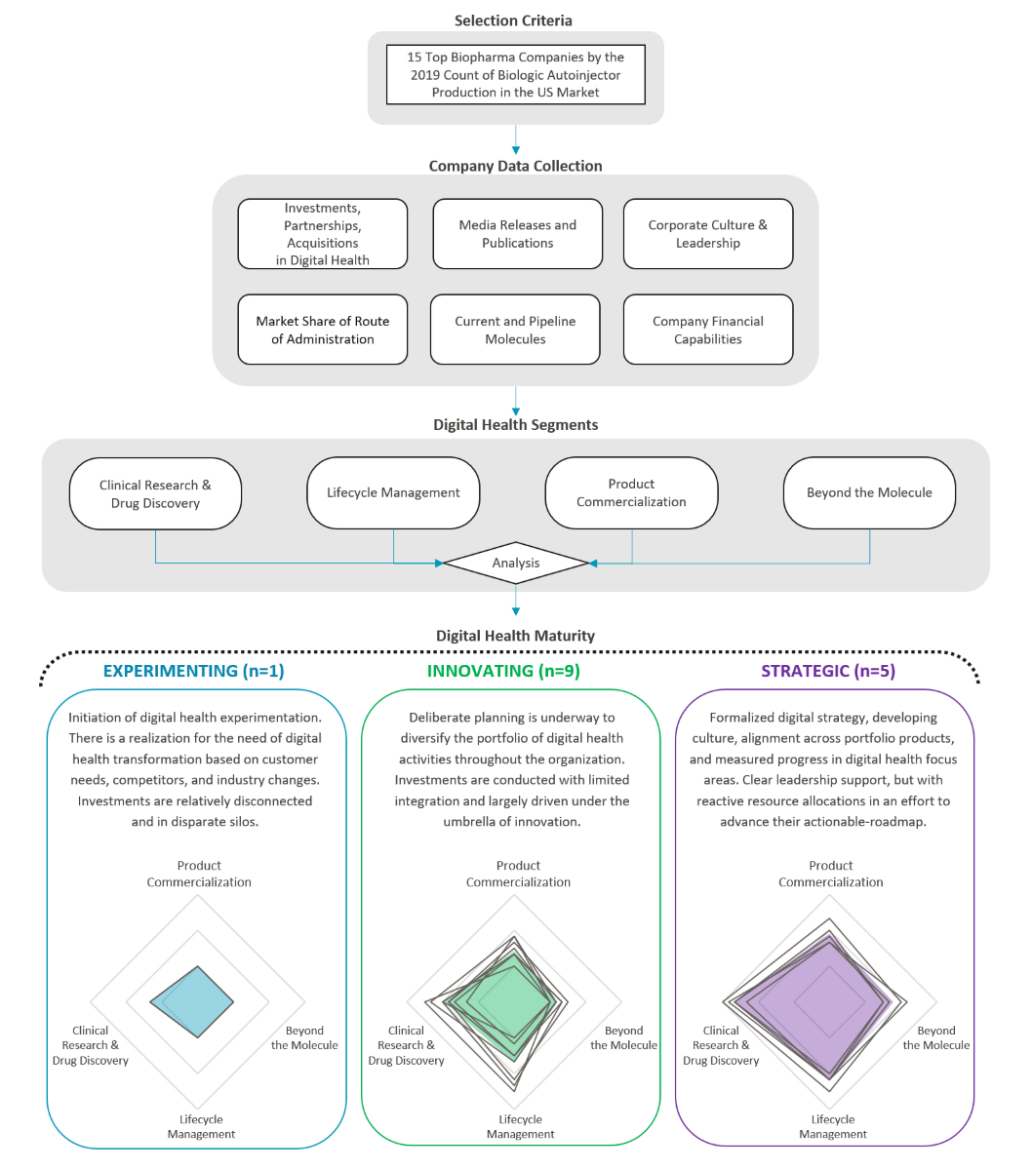
Framework for biopharma digital maturity assessment. This figure demonstrates biopharma’s overall approach toward digital health based on a segmentation analysis covering clinical research and drug discovery, lifecycle management, product commercialization, and beyond the molecule. Companies were classified within three distinct digital health maturity categories. Radar charts show individual company ratings for each segment, and shaded regions represent the average for each category.

We define digital health maturity as biopharma’s organizational transformation by adopting digital health technologies, real-world evidence generation, digital-first leadership, and alignment of the product portfolio strategy. To assess the forces driving the next generation of autoinjectable devices, we quantified the digital health maturity of the top 15 biopharmaceutical companies in the US biologics autoinjector market. We conducted a detailed analysis of each company to evaluate their digital health activities across the following four segments: Clinical Research and Drug Discovery, Lifecycle Management, Product Commercialization, and Beyond the Molecule (Table 1). This framework for rating each biopharmaceutical company’s maturity used qualitative and quantitative factors as described in Multimedia Appendix 1. The information from this maturity rating for each company is drawn from publicly available sources as of October 1, 2020, including US marketed and pipeline molecules, digital health–related strategic investments, partnerships and acquisitions, estimated spending committed to digital health endeavors, senior leadership’s experience, and public statements addressing their digital health vision. A combination of public and private databases was used, including EvaluatePharma. To the degree that a biopharmaceutical company may have additional digital health initiatives in the abovementioned four segments that are not disclosed to the public, the company’s maturity rating may be underestimated. The digital health maturity for each biopharmaceutical company is represented by a single number (between 0 and 1), which is the sum of each company’s segmentation scores (0-20) normalized to the maximum value. The 15 companies were grouped into one of the following three categories: experimenting (bottom one-third), innovating (middle one-third), and strategic (top one-third), as shown in Figure 1.

**Table 1:** Four digital health segments used to evaluate biopharmaceutical company maturity.

Digital health segments	Definition
Clinical research and drug discovery	Process improvements in clinical research and drug development enabled by digital health to realize clinical benefits.
Lifecycle management	Continuous monitoring and improvement of the product or service through real-world evidence generation to meet the needs of the end user until product end of life.
Product commercialization	Digital health extensions of the molecule’s capabilities outside of pure pharmacokinetic or pharmacodynamic impacts that increase the value of the molecule.
Beyond the molecule	Hardware or software solutions that provide therapeutic benefits independent of the molecule.

## Key Themes of Biopharma’s Digital Health Maturity

There is a clear differentiation among biopharma’s digital health maturity, as seen by the radar charts in Figure 1. Each chart’s center is skewed toward clinical research and drug discovery, demonstrating an increasing focus from companies who are adapting new technologies to their drug development processes. As we move from left to right across the categories, the area of the shaded region increases, representing expanding levels of digital health maturity across all four segments. It is interesting to note that where the experimenter focuses primarily on clinical research and drug discovery, innovators expand their interests to include lifecycle management with limited focus on product commercialization. At the same time, strategics show substantial activity in both product commercialization and beyond the molecule segments.

**Figure 2:**
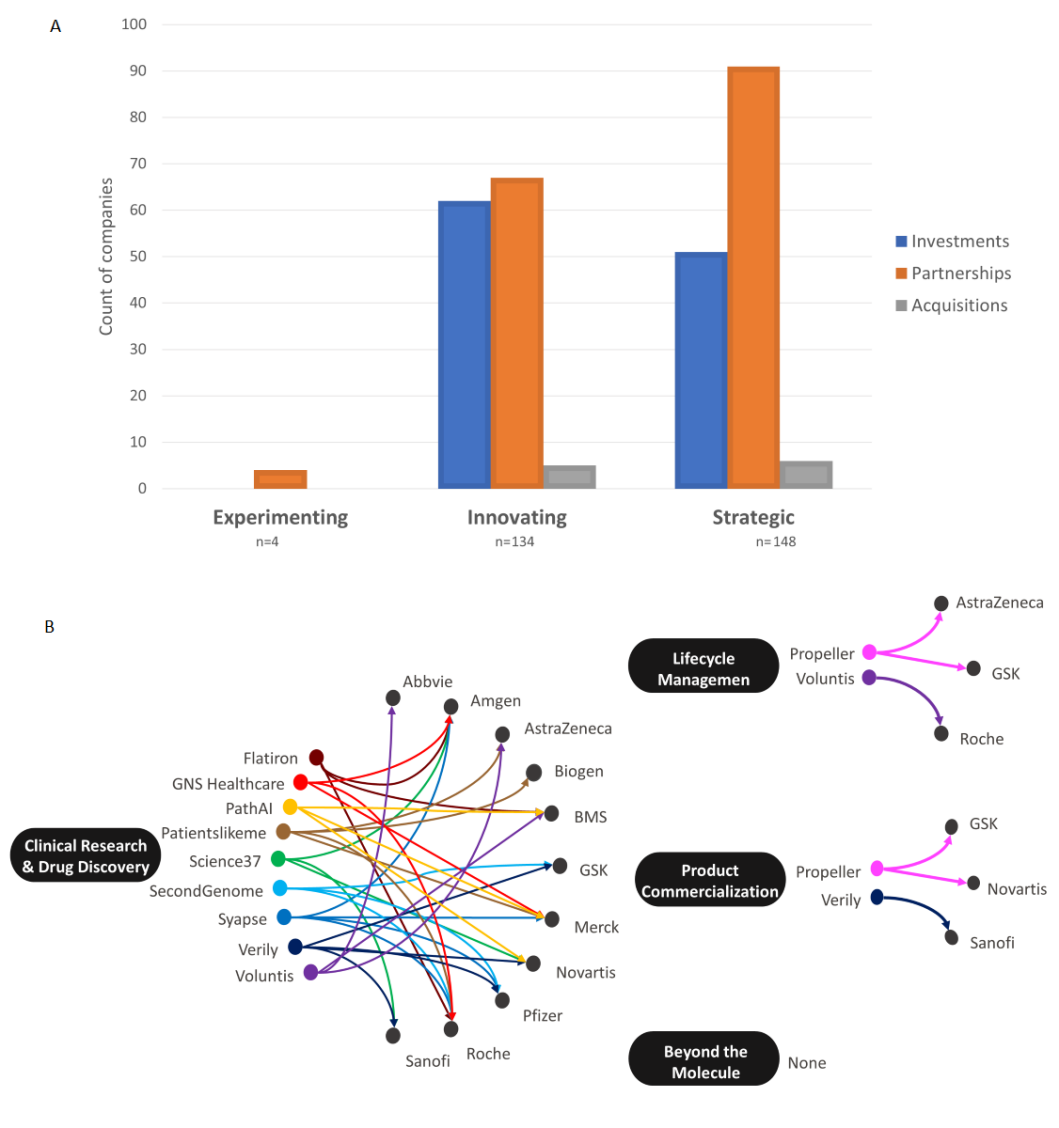
(A) Distribution of biopharma’s digital health partnerships, investments, and acquisitions from 2010 to 2020. Investments included direct investments from biopharma or a subsidiary arm of the parent company. Partnerships are the preferred format for achieving digital health maturity. (B) Digital health companies with at least three different biopharma interactions (eg, investment, partnership, and acquisition) were plotted against the four digital health segments. Currently, clinical research and drug discovery digital health companies encapsulate the largest segment of investments or partnerships from biopharma. None: the analysis did not return any digital health companies focusing on the beyond the molecule segment, which met the minimum criteria of at least three different biopharma interactions.

This analysis found 286 digital health companies working with biopharma. Considering the nature of biopharma’s engagement with digital health, we classified the overall approach taken by companies in each category based upon investments, partnerships, and acquisitions (Figure 2A). Currently, biopharma is heavily vested in forming partnerships in digital health. However, there is a clear distinction with innovators and strategics who have a greater appetite for risk through investments and acquisitions. Across the board, biopharma’s acquisitions have been limited so far, which may be due to a lack of perceived value or the complexities in integrating vastly different organizational cultures. Today, partnerships are the desired format for achieving digital health maturity. Figure 2B highlights prominent digital health companies defined as having a relationship with at least three or more of these biopharmaceutical companies. It is important to note that the four segments had varying trends, with a heavier focus on clinical research and drug discovery.

Biopharma’s core business is in clinical research and drug discovery, and as such, there is a primary focus on digital health efforts in this segment. Clinical trials are becoming increasingly complex and biologics are vastly more expensive to discover, so biopharma is using artificial intelligence to reduce attrition rates and research and development expenditure, and the vast data can accelerate the understanding of disease pathology and identify new drug targets and candidates [29-31]. Biopharma is turning toward digital health to not only improve the data collected from enrolled participants but also increase patient recruitment and retention (the largest cost driver of clinical trials) by engaging with patients through social media platforms or online health communities [32-35]. The premise is that by using digital health, they may shorten the time spent in clinical research while also amassing previously unattainable real-world data. Digital biomarkers will serve to generate novel data endpoints outside of traditional clinical environments and expand insights directly from the patient’s home [8]. Biopharma will need to determine which digital biomarkers are valuable and how to integrate them into research. The overall digital health premise of data generation at the patient’s home is highly attractive [36]. Our data reinforce this point as all companies regardless of their maturity category have applied digital health to their clinical research and drug discovery [37].

The second largest segment is lifecycle management where its implementation varies across companies as influenced by their digital health maturity. Its most basic manifestation consists of packaging or a companion app, while higher sophistication levels have been demonstrated through connected DDCPs [38]. These products can be considered to provide support for biopharma’s drugs and often fall in line with their therapeutic portfolios, generate real-world evidence, and aid in gathering novel data sets to differentiate and extend the longevity of their molecules. One of the key themes was user-focused mobile apps to empower patient disease self-management; however, a high churn rate remains a strong barrier to achieving the desired health outcomes [39]. Our findings clearly demonstrate that innovating and strategic companies have made considerable inroads with the application of digital health to lifecycle management.

As companies continue to commercialize their drug products, few are proactively designing these products with integrated digital capabilities. The minimum design for these devices is Bluetooth connectivity to a patient-facing mobile app. This connected DDCP can act as an adherence measure, allowing patients to keep a record of their medication use and share their data with their providers [40,41]. Some companies have taken a further step by creating entire platforms for their devices, including daily predictive forecasts and integrated and streamlined communication with health care providers and support programs. Expanding beyond this includes integrated sensors and apps enabling drugs to aid in chronic disease management, which can collect a variety of data from general adherence to pharmacokinetic and pharmacodynamic data that could better inform patients and their providers about usage behaviors to optimize drug adherence and treatment [9,42,43]. The next step will be fully connected DDCPs dispensed from the pharmacy, which has been seen with bioingestible sensors in oral medications and connected inhalers [44,45]. Both innovators and strategics have achieved limited product commercialization and have not yet fully explored how user error could impact collected data in chronic disease management [46].

Of all the segments evaluated for biopharma’s digital health maturity, beyond the molecule is the least explored, demonstrating the reservations toward digital therapeutics. Multiple companies in the innovating and strategic groups have partnered with a digital therapeutic company. As seen in Figure 2B, no digital health company has succeeded in attracting multiple biopharma interests in beyond the molecule solutions compared to those focused on other segments. Digital therapeutic companies’ narrow therapeutic focuses may not align them across multiple biopharma pipelines at this time. Other areas of emerging focus in the beyond the molecule segment include gamification technology and virtual reality that could offer novel therapeutic treatments [47-50]. The digital therapeutics space is an area that will blossom; however, the push to embrace a beyond the molecule business model is in its infancy today and future growth is largely expected to be driven by strategics and a few innovators [51].

Biopharma’s internal cultural dynamics can influence an organization’s digital health maturity. To better understand internal leadership culture, individual digital health leadership industry backgrounds were aggregated across the previous 15 years based on their disclosed roles on a professional social media platform (ie, LinkedIn). As biopharma’s maturity increased, leaders had more diversified backgrounds and companies relied less on promotion from within the biopharmaceutical industry (Table 2). This clear correlation may attribute low digital maturity to a lack of outside novel perspective. One of the core limiting factors encumbering biopharma is the lack of personnel with formal education and training in digital health. Relatively few programs are currently focusing on digital health training, as seen by the lack of standard practices and education [52]. As such, there is a definitive digital health skills shortage across biopharma, and this is demonstrated by the largest sector of personnel being internally promoted to digital health–focused divisions, regardless of the company’s maturity level.

**Table 2:** Digital health leadership backgrounds.

Industry^a^, (n=235)	Experimenting	Innovative	Strategic
Biopharma	76%	53%	44%
Health care	0%	10%	12%
Marketing	0%	2%	4%
Medical devices	0%	3%	2%
Consulting	3%	13%	12%
Education	14%	6%	2%
Finance	0%	1%	2%
Information technology	7%	4%	6%
Others^b^	0%	8%	16%

^a^Internal biopharma digital health leadership backgrounds have been evaluated across the three maturity categories. The sample size includes 235 digital health employees that hold an executive, head, or director level position.

^b^“Others” include: Retail, Hospitality, Food & Beverage, Telecom, Media, VC, Government, Entertainment, Utilities, Staffing, Insurance, Renewable, Travel, Apparel, Cosmetics, Research, Consumer Goods, Law, and Farming.

## Evolving Biopharma Pipeline Driving Innovation in Autoinjectable Devices

**Figure 3:**
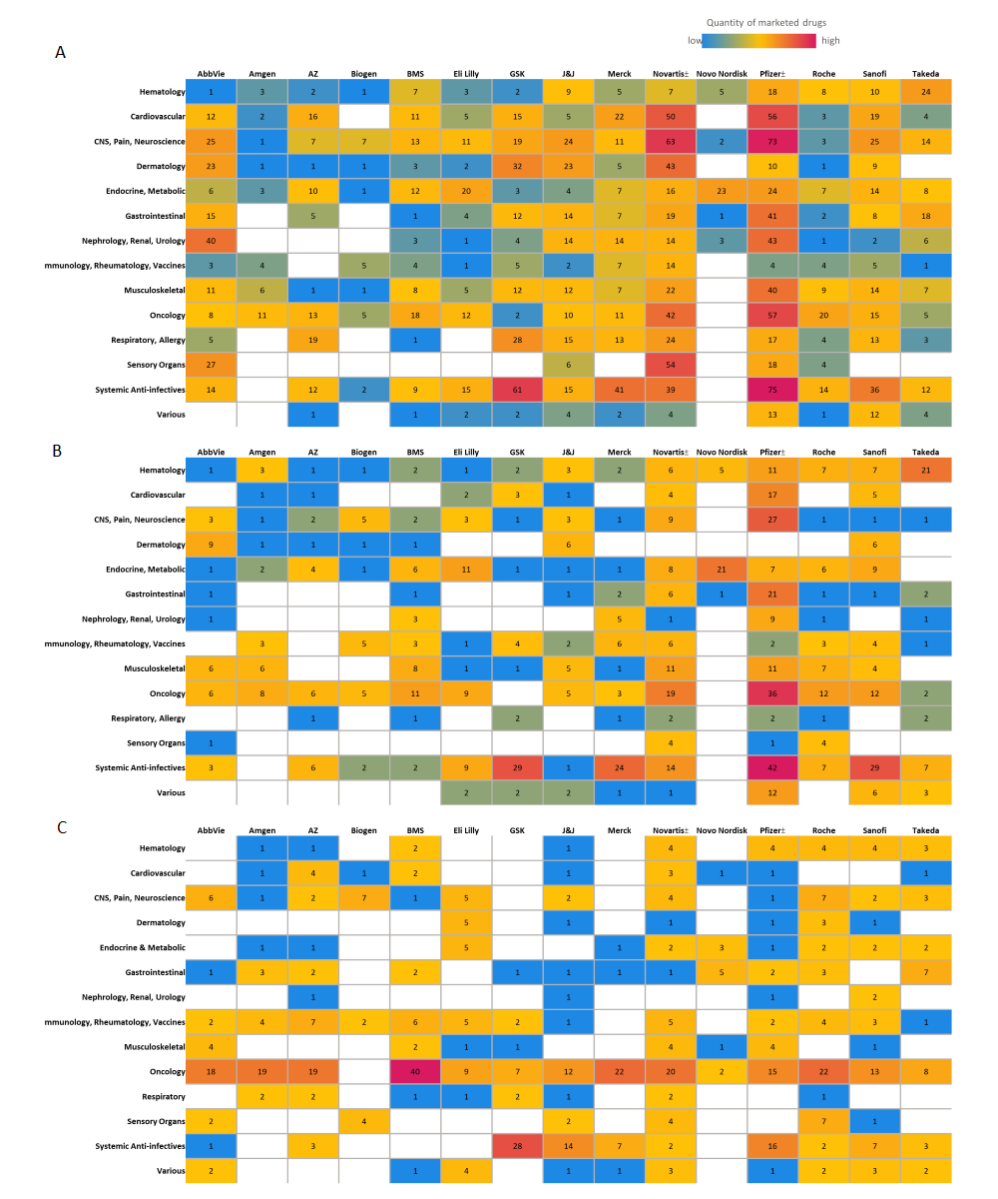
Biopharma portfolios and pipeline by therapeutic areas. Heatmaps showing (A) marketed molecules across all routes of administration, (B) marketed injectable molecules, and (C) phase I to III pipeline molecules proposed for injectable administration. Biopharma pipeline assessment for the US market was conducted via the EvaluatePharma database as of October 1, 2020. Therapeutic areas defined in this paper were standardized across each biopharmaceutical company. For Novartis and Pfizer, generic manufacturing has been included in the count.

Many digital health forays have been focused on clinical research activities. Figure 3A-C presents heat maps showing the number of drug molecules by biopharmaceutical companies and therapeutic categories based on their US primary indication for (1) currently marketed molecules, (2) currently marketed injectables, and (3) active injectable drugs in the pipeline. When comparing current marketed injectables (Figure 3B) to pipeline injectables (Figure 3C), there is a noticeable shift across the majority of companies toward autoimmune conditions and oncology.

In the autoimmune category, the major biopharmaceutical companies have increased their deal making with respect to acquiring products to fill their pipelines. Both in-licensing and out-licensing activities have shown notable increases in the last few years (2013-2018) [53]. Biopharmaceutical companies licensing-in products are paying more than twice as much for new autoimmune products in the recent period (ie, 2013-2017) than they did in the previous 5-year period (ie, 2008-2012) [51].

Oncology is the leading therapeutic category in the injectable pipeline. Oncology deal-making saw an increase of 142% in the period of 2013 to 2017, with 643 deals compared with 266 deals in the period of 2008 to 2012 [53]. Oncology is the major therapeutic focus in the injectable pipeline for 12 of the 15 companies evaluated in this study. Interestingly, research is being focused on subcutaneous delivery of oncology products, with trastuzumab researched extensively [37,53-55]. With the discovery of more biologic therapeutic agents, we see more cancer patients being treated at home rather than in controlled inpatient settings. Moving forward, the administration of advanced biotechnology-derived agents will be more prevalent in the home environment. Oncology specifically offers tremendous market potential for drug products engaged with digital health technologies to address various unmet needs for patient care [56]. The expanding oncology pipeline can be combined with novel research approaches using site-less trial designs to study and deliver effective therapies to otherwise high-value therapeutic markets [57].

## Framework for Next-Generation Connected Autoinjectable Devices

**Figure 4:**
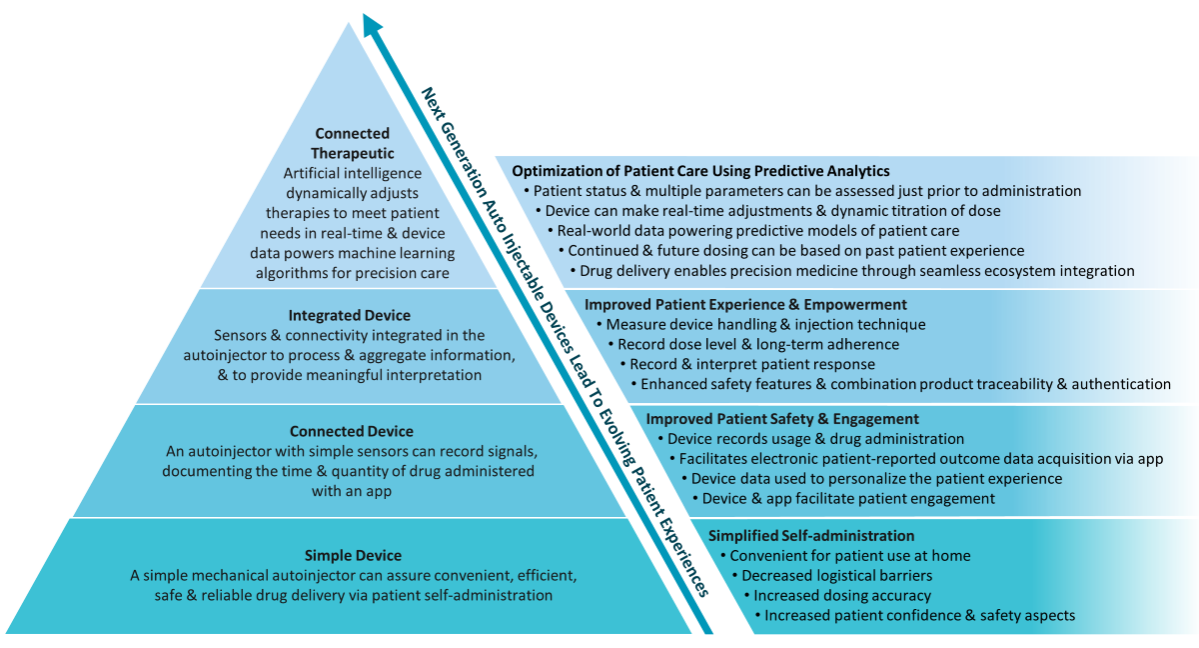
Framework for next-generation connected autoinjectable devices. This framework for next-generation connected autoinjectors demonstrates the technological hierarchy of design (left) that transitions the simple device to a connected therapeutic. Through this design evolution, the autoinjector shifts to become the focal point of decentralized precision care for many chronic conditions, powering artificial intelligence disease management systems that impact overall patient care (right).

The framework for a connected autoinjectable device is multifaceted but resides in addressing today’s unmet need for patient self-administration in the home. Figure 4 highlights the autoinjector’s evolution trajectory from a simple device to connected therapeutics. In its most basic form, the autoinjector is designed to facilitate patient self-administration via a simple process, while retaining patient convenience and safety. This exchange does not elicit measurable objective outcomes for stakeholders, such as patient adherence, injection technique, and patient-reported outcomes. Moore law, combined with advancements in digital health applications, is now enabling the development of connected or integrated autoinjectors to varying utilization levels [58]. These sensors and communication modules not only elevate the safety aspects of the device (eg, temperature monitoring, authentication tags, recalls, and tampering alerts), but also track measurable patient outcomes (eg, dose-level adherence, injection technique, side-effects, and therapeutic outcomes) tethered to a mobile app or hub for data acquisition, transmission, and analysis. These connected and integrated autoinjectors can now enhance patient engagement and guide therapeutic decision making with objective data outputs. A connected therapeutics product then shifts the data value away from the clinic and to the home, when high-resolution objective data outputs from connected or integrated autoinjectors are captured to power machine learning predictive models to reliably inform real-time care decision making. The autoinjector then transitions to become the focal point of decentralized precision care for many chronic conditions, enabling artificial intelligence disease management systems to track or predict patient outcomes (eg, therapeutic outcomes, major events, experiences, and side effects) both individually and at the population level. Overall, connected therapeutics is the highest evolution of the connected autoinjector, which is represented by the pyramid peak in Figure 4.

As previously identified, biopharma will be the leader in championing this paradigm shift toward connected therapeutics, but internal culture and leadership will likely dictate adoption over the coming years. For autoinjectors to no longer be viewed as simple devices facilitating drug administration, but as an enabling technology for decentralized precision care, a shift in mindset is required. To create this shift, biopharma will need to foster an innovation culture, achieve digital health workforce diversity, and leverage partnerships outside the biopharmaceutical industry. We have demonstrated a considerable discord in digital health leadership across all three maturity categories in our analysis. Most evident are the strategics with the most diversified leadership, encapsulating outside novel perspectives and demonstrating the greatest appetite for external partnerships across the four digital health segments, which sets them apart from experimenters and innovators. As such, more mature companies will likely extend the value proposition of connected autoinjectors and expand to connected therapeutics through funding research and engaging providers and payors for market-shaping strategies. In comparison, it is expected that experimenters and innovators will focus on connected autoinjectors for their current products’ lifecycle management as they determine how to achieve broader market differentiation until their leadership envisions a digital health strategy.

An evidence-based approach, utilizing interdisciplinary teams of clinical, engineering, economic, and behavioral science experts, will be critical for demonstrating the feasibility of next-generation connected autoinjectable devices [58]. Our evidence generation value proposition is however not necessarily novel [59]. The connected inhaler space has shown multiple successes and generated considerable evidence as a model to follow, but the application of the body of evidence is narrow owing to the few therapeutic categories (eg, asthma and chronic obstructive pulmonary disease) that inhalers address [60]. In comparison, the autoinjector market is much more differentiated across a whole spectrum of conditions, demanding an evidence generation process that is specific to each patient population, the disease condition being addressed, and the pharmacotherapy [61]. This will be a significant endeavor as our analysis has demonstrated a growing pipeline of therapies focused on chronic conditions using injectable molecules, in addition to the likelihood of oncology becoming a growing market for home self-administration [62]. While a focus on adherence is currently the simplest business case to encourage evolution of the autoinjector toward connected therapeutics, biopharma will also need to demonstrate improvements in clinical outcomes via providers, increased economic efficiencies via payors, and patient satisfaction and usability via sustained levels of engagement [63]. Additional concerns will be on how connected therapeutics will integrate within a broader health care ecosystem, embracing remote diagnostics and digital therapeutics–augmented treatments, in order to enrich actionable data sets and reduce data silos that have historically led to a poor uptake of digital health interventions [64].

## Conclusion

Next-generation autoinjectable devices will play an important role in implementing biopharma’s digital health approach to the biologics market. Our analysis demonstrated considerable biopharma maturation differences with digital health. In the coming decade, biopharma will need to design a strategic and methodological pathway to embed digital health as a key corporate cultural aspect in order to succeed. Utilizing digital health, connected therapeutics will allow biopharma to achieve a closer relationship with patients in the home and with providers, as our framework establishes. With autoinjectable devices enabling home self-administration and connected therapeutics powering the delivery of precision care at home, biopharma will need to drive innovation in autoinjectors to achieve the full potential of biologics.

## Appendix

Multimedia Appendix 1 Qualitative and quantitative factors used to establish the maturity rating for each biopharmaceutical company.

## Abbreviations

DDCP: drug device combination product
