# Supplementary material for: Digital Health Integration Assessment and Maturity of the United States Biopharmaceutical Industry: Forces Driving the Next Generation of Connected Autoinjectable Devices
Source: JMIR Mhealth Uhealth. 2021 Mar 18;9(3):e25406. doi: 10.2196/25406 (PMC8088878; doi:10.2196/25406)
Supplement: Multimedia Appendix 1 [file mhealth_v9i3e25406_app1.docx]

## **Multimedia Appendix 1. Qualitative and quantitative factors used to establish the maturity rating for each biopharmaceutical company.**

| Rating | Description | Qualitative | Quantitative |
| --- | --- | --- | --- |
| 1 | Minimal activities, clearly naïve | * Naïve or novice;  * Minimal activity & effort not focused;  * No strategic theme;  * No cohesive digital health strategy or vision communicated publicly. | * No or little activities;  * Initial investment or partnerships explored;  * No tangible results or products;  * Very limited financial investment. |
| 2 | No formalized strategy, making Initial investments | * Beginner;  * Digital products in development, but no or little tangible results or products;  * Too early to see strategic themes, but some strategic elements present;  * Strategic themes developing, but not a structured strategy. | * Few activities;  * Starting investments & partnerships;  * A few tangible results or products;  * Limited financial investment. |
| 3 | Emerging strategy, initiating multiple partnerships | * Experienced;  * Emerging & substantial products in 1 or 2 therapeutic areas;  * Strategic themes are emerging with digital strategy for 1 or 2 categories;  * Some strategy for digital health beyond core business (e.g., incubator program). | * Moderate activities;  * A few investments & partnerships;  * Tangible results or products in 1 or 2 focused areas;  * Substantial & growing financial investments. |
| 4 | Strategy developed, implementation in progress | * More advanced;  * Demonstrated digital health activity beyond core business;  * Articulated strategic themes, but may appear uncoordinated at times;  * Digital health strategies defined but needs focus & a plan for action. | * Many activities;  * Multiple strong investments & partnerships across several firms;  * Several tangible results or products;  * Strong financial investments. |
| 5 | Strategic, formalized, publicized, and integrated plan | * Very advanced;  * Digital health products & offerings across many areas & beyond the core business;  * Strong strategic themes stated;  * Strategic plan is focused & clear. | * Extensive activities;  * Very strong investments &  partnerships across diverse industries;  * Successful tangible results or products;  * Very strong financial investments. |
